# Sustainable sojourns: Fostering sustainable hospitality practices to meet UN-SDGs

**DOI:** 10.1371/journal.pone.0307469

**Published:** 2024-07-24

**Authors:** Jing He, Umer Zaman

**Affiliations:** 1 School of Culture and Tourism, Shanxi Finance & Taxation College, Taiyuan, China; 2 Endicott College of International Studies, Woosong University, Daejeon, Republic of Korea; Guangxi Normal University, CHINA

## Abstract

This research discusses the significance of environmental transformational leadership (ENTL) in the creation of energy-saving sustainable behaviors (EESB) among employees in the tourism and hospitality sector of China. The method is consequently a quantitative analysis, where the mediating effect of green intrinsic motivation (GNIM) and green passion (GRP), and the moderating role of green altruism (GNA) are examined to understand their influence on the relationship between ENTL and EESB. The data were gathered from multiple hotels in major Chinese cities, with the use of a structured questionnaire. The study shows that ENTL has significant effect on EESB, with GNIM and GRP serving as mediating factors. In addition, GNA was shown to have been able to boost the effects that ENTL has on these mediators. The findings are indicative of the vital role of leadership in promoting responsible practices within the tourism and hospitality sector, and towards the attainment of the UN Sustainable Development Goals. This research not only fills the gaps in the existing literature that primarily focuses on developed economies but also provides policy makers and business leaders with practical solutions for enhancing the sustainability in emerging economies.

## 1. Introduction

Climate change constitutes an existential risk requiring immediate action to safeguard endangered ecosystems and human societies [1, 2], consistent with the United Nations’ Sustainable Development Goals (UN-SDGs), notably Goal 13- climate action. The contributions of both the global initiatives and individual actions in creating environmental sustainability cannot be overemphasized. Therefore, the role of minimizing carbon dioxide emissions which majorly involves personal choices is crucial for the proper functioning of our planet [3]. Tourism and hospitality industry is one of the most important industries in the world economy since it is the source of employment, economic growth and cross cultural exchange [4]. However, it also has a significant environmental cost, main effects of which are resource use, waste production, and emissions of greenhouse gases [5]. This places the industry in a strategic capacity, where it has not only the opportunity but also the obligation to be a pioneer in sustainable development. Sustainable practices promotion allows the tourism and hospitality sector to provide a major contribution to the overall achievement of the UN-SDGs. This sector might act as a guide to other sectors, showing that one does not have to compromise the environment for economic growth. The implementation of sustainable strategies in this sector not only helps to reduce the negative effects on the environment, but also makes this industry more attractive to consumers who are sensitive to the environment. Thus, there is a need for research to explore the factors that influence the sustainable actions among employees in the industry and, therefore, encourage the culture of sustainability.

Our consumption behavior in the globe is the major culprit of greenhouse gases emissions (almost 72%) [6] that account for more than half of the global total, encouraging shifting to responsible consumption and production as highlighted in Goal 12 [7]. The narrative goes beyond global policies to individual behaviors in moderating climate impacts. Although it is crucial to transition to renewable energy, addressing our consumption behaviors equally matters for environmental sustainability [8, 9]. In advanced economies, organizations are moving to the carbon zero stage; yet, encouraging employees to adopt sustainable consumption lifestyles will be crucial for overall environmental responsibility [10, 11]. Employees’ personal consumption behaviors have a strong bearing on an organization’s overall green footprint which highlights the need to encourage energy conservation and sustainable consumption at the level of the individual as a means of contributing to the industry’s efforts in reducing environmental impact [12].

Human behavior in organizational sustainability is determined by both organizational and individual factors [13–16]. The literature reveals that transformational leadership affects the innovation of sustainable employee behaviors, thus; a leader who is environmentally transformed may induce energy conservation practices among staff members [17]. In today’s rapidly evolving tourism landscape, recognition by esteemed industry bodies such as the World Travel Awards plays a pivotal role in setting benchmarks for excellence and innovation. The 2023 award for the World’s Leading Tourist Board is a testament to the dynamic strategies and practices that define current trends in global tourism [18]. Our study draws upon these developments to frame our investigation into environmentally-focused transformational leadership (ENTL) within the hospitality sector. To strengthen the theoretical base, we explore deeper themes of the origins of transformational leadership, especially its environmental dimension. Through the conceptualization of ENTL as a leadership style that concurrently motivates, inspires, and strategically emphasizes sustainability, we link our study to contemporary environmental management theories [19]. The elaboration of this definition assists us to build up the analysis that we will provide later in the manuscript. It creates a strong foundation that supports the empirical investigations described in detail. Practical examples strongly reinforce the critical role of transformational leadership and personal motivation in effecting sustainable behaviors within organizations. As an example, Siemens AG’s move to sustainability was propelled by its CEO’s passion to be an environmental steward, subsequently; the company adopted energy-saving technologies and a general policy of reducing waste. Under this leadership, Siemens AG began a journey to become carbon neutral by 2030 and as a result, there was a 30% reduction in carbon emissions within three years since the implementation of the sustainability strategy [20]. In a parallel way, Kimpton Hotels & Restaurants demonstrates that incorporating incentives can result in organizational change through sustainable practices. Kimpton launched the ’green rewards’ program to motivate employees to conduct eco-friendly actions such as conserving energy and engaging in recycling activities. This not only supported a considerable reduction in the firm’s environmental footprint but also improved workers’ involvement and satisfaction, therefore demonstrating the power of putting sustainability at the core of corporate culture [21]. These cases illustrate the transformational nature of leadership and the importance of intrinsically green motivation in creating an organizational culture that champions sustainable practices. Furthermore, personality traits which include green intrinsic motivation (GNIM) and green passion (GRP) are crucial as mediators in promoting sustainable behaviors stressing the need for investigation into how these attributes mediate the relationship between ENTL and energy efficiency in particular [22, 23]. The basis of a separate treatment of GRP and GNIM as different variables is their specific psychological nature and the impact of these variables on sustainable behaviors, which may not be obvious. GRP is conceptualized as a feeling of emotional attachment and affection toward environmental sustainability [24]. The existence of this feeling creates a motivation for individuals to practice sustainability in their daily life. Conversely, GNIM is defined as the internal satisfaction that employees have from participating in environmentally sustainable activities, regardless of the incentives attached [25]. Through the separation of affective (GRP) and personal motivational (GNIM) components, our research provides an acknowledgment of the intricate relationship between emotions and personal values within the shaping of sustainable behaviors. This difference permits us to accurately quantify how particular components of personal motivation and emotion affect the introduction of environmentally-friendly practices, which in turn provide a deeper understanding of the mechanisms through which ENTL promotes employee sustainable behavior. This study further investigates the role of green altruism (GNA) in augmenting the effects of ENTL on employees’ sustainable behaviors through GNIM and GRP [22]. Through studying the mediating roles of GNIM and GRP, and the moderating role of GNA, this study aims at revealing deeper mechanisms of interaction between transformational leadership and personal values to stimulate energy-efficient sustainable behavior (EESB) within the employees of tourism and hospitality sector.

Specifically, Asia-Pacific is blamed for contributing more than half of total CO_2_ emissions globally. In this regard, more than 50% of total CO_2_ emission from Asia-Pacific comes from electricity, as per the report by Asian Development Bank (ADB) [26]. Given the energy sector’s role in this consumption and its contribution to greenhouse gases, emphasizing Goal 7- affordable and clean energy is also vital. The sector’s significant role, emitting 35% of global greenhouse gases, underlines the priority of both policy and individual behavior shifts. Significantly, China becomes an important player, with 59% of the regional and 28% of the global emissions, emphasizing its central role in the global environmental sustainability process [27]. China’s commitment to carbon neutrality by 2060 implies a huge leap towards substantial change, which requires a combination of policy reforms, technological innovation and alteration of the infrastructure and individual conduct [28].

China’s shift towards sustainability and incorporating it in every development aspect, from leading in clean energy to the traditional sectors, is vital. The 2060 carbon neutrality target of the country shows the important balance between ambition and real action towards climate change. Based on the current aggregation of nearly 200 climate pledges, most anchored in the Paris Climate Agreement, global temperatures are still projected to escalate by an alarming 2.7 degrees Celsius by the century’s end. Notably, China’s pledge alone has the potential to shave this estimate down to 2.4 to 2.5 degrees Celsius, a testament to its profound impact on global carbon trajectories [29], however, this is still above the safe limit of 1.5°C which is dangerous, urging the world to action towards carbon neutrality by 2050. Given its status as the world’s biggest emitter, the actions of China have serious implications for the rest of the world, acting like a blueprint for the decisions of other countries.

This study attempts to address important gaps in the literature on the environmental sustainability, and in particular, how personal behaviors within tourism and hospitality sector, influence sustainability practices. We specifically examine the role of personal factors such as GNIM and GRP and how ENTL which focuses on the environmental aspects promotes sustainable behavior among hospitality employees. It provides implementation of SDG 12 and shows that leadership is important to the promotion of sustainability. Our exploration of GNIM, GRP, and the neglected human value of GNA, focuses on how they interact and exert influence on workplace sustainability, and through which strategies can help employees in tourism and hospitality carry out sustainable behavior across different situations.

Moreover, when we place our research in this Asia-Pacific context, the region contributing immensely to global CO_2_ emissions, our study draws attention to the role of leadership, personal inspiration, and motivation for achievement of environmental sustainability in emerging economies. Thus, the paper not only addresses the gap in the literature on sustainable behaviors within organizations but also provides useful information for companies in the Asia-Pacific and elsewhere that want to improve their ecological footprint by using effective management and employee engagement approaches. This focus calls for a combination of ENTL with personal factors to secure the likely triumph of the organization when it comes to sustainability, contributing to the broader goals of environmental stewardship and climate change mitigation. Particularly for China, with the goal of net zero carbon by 2060, our study underlines the necessity of adopting sustainable consumption and production of energy (aligned with SDGs 7 and 13). We investigate ENTL in China’s tourism and hospitality sector with an aim to provide insight on the promotion of sustainable behaviors in the emerging economies, addressing the current literature gap in the sustainability of the developing countries. On a further note, the focus of this research offers unique implications guidance on how ENTL is a clear and effective driver of the organizational culture towards sustainability. In addition, personal factors such as GNIM, GRP, and GNA are addressed as they are the means to have a workforce that is ready to see to environmental stewardship. Lastly, the novel challenges and the opportunities which remain from the Asia-Pacific, together including the tourism and hospitality industry, will be explored here under as the main field high-risk area to the resource conservation with sustainable approach.

## 2. Theory and hypotheses

Social learning theory is a strong foundation to comprehend the employees’ EESB towards their organizations in terms of ENTL, thus providing a strong conceptual framework [30]. This theory primarily supports that individuals learn from others and what they do and think really matter for those who are led or supervised by them [31]. For instance, ENTL as part of his/her role practices that are embedded with environmental sustainability [32]. Such kind of leader inspires and leads subordinates through eco-conscious behaviors such as energy conservation including environmental concerns into decision making process [17]. According to social learning theory which forms the basis for this research, these behaviors could be imitated by workers from the leaders [33, 34]. The theory argues that if employees view the leader treats natural resources carefully, it can become a way of changing their own minds about doing things. In addition, it is expected they will adopt similar measures becoming more environment responsible thus preserving the nature [35]. Essentially therefore, social learning theory gives a good theoretical basis to understand how ENTL can affect EESB within organization [36]. Acting as exemplars of sustainable conduct on one hand, while implementing principles of social learning on the other hand, allows ENTL to effect behavioral changes among employees leading to improved EESB so it goes beyond merely setting an example.

The relationship between the employees’ EESB and ENTL may be thoroughly examined using social learning theory. Within this context, ENTL guides organizational values and goals regarding environmental issues besides serving as a powerful model for employees’ emulation [37]. Furthermore, unlike other forms of transformational leadership, ENTL is a specific type that stresses environmental rather than mere economic or efficiency considerations when deciding on its priorities [38]. Leaders who practice environmentally conscious transformational leadership tend to embody an environment vision that is both well-articulated and convincing; embracing green values; showing genuine commitment to environmental preservation [38]. Such leaders’ behavior as leaders and managers is not limited to verbal communication, but they consistently indicate preference for green alternatives at the expense of immediate economic gains [36]. These are some of the actions that such leaders take which becomes a source of learning for their subordinates.

ENTL becomes unique due to valuing the environmental parameters by involving them as decisive factors instead of measuring the economic ones or indicators of efficiency [37]. In this context, leaders are the ones promoting responsible ways of life, preaching the urgency of environmental stewardship and finally motivating others to embrace the same convictions and habits. This leadership style adopts the core UN SDGs, especially 7 (sustainable energy), 12 (responsible consumption), and 13 (climate action), by facilitating a culture that focuses on and incorporates sustainability [39]. We base our hypothesis on the fact that employees copies the environmental commitments that are mirrored by their leaders whilst at work through the observational learning which is the key mechanism used in the social learning theory [40]. This relationship evidences the fact that ENTL can bring about a transformation in an organization’s environmental culture, and thus, employees can become more committed to certain environmental objectives and sustainability practices which could be established. Hence:

**H1:** Energy specific sustainable employee behavior can be predicted by environmentally focused transformational leadership

The intrinsic motivation of employees particularly GNIM is a crucial factor in enhancing green agenda within organizational settings [41]. Deci and Ryan [42] definition of intrinsic motivation is adapted here in an environmental context to define GNIM as a psychological process that internally motivates employees to fulfill tasks with an environmental conscientiousness manner. Intrinsically motivated employees are satisfied internally thus motivated by individual desire rather than financial rewards alone; these workers go beyond monetary gains using modern equipment or tools that protect nature [22]. Such practical attitudes stem from inner control over one’s own life besides concern for nature making staff respond to the environment naturally [43]. According to Wu, Chen [44], employees’ GNIM can help shape their behaviors at work. In light of this, GNIM entails doing everything possible in order to minimize environmental impacts thereby enhancing businesses sustainable development. To summarize, organizations that have employees who are intrinsically motivated to conserve nature or protect the ecosystem are committed to preventing damage to the natural environment that may arise from individual or corporate decision-making [45].

Nevertheless, promoting this sort of motivation and nurturing a culture of sustainability to a large extent depends on the leadership role within an organization [46, 47]. Instead, ENTL plays a major part since several research studies have highlighted that green transformational leaders have a great influence on employees as far as eco-friendly and sustainable approaches are concerned [48, 49]. Through inspiration by their vision, GNIM can be stimulated by ethical conduct as well as sustainable practices. Leading from the front, they embody the commitment to environmental sustainability that goes beyond mere talk. In doing so they can create organizational cultures in which financial gains are not motivators for employees but shared values on environmental protection/sustainability [50, 51]. Therefore, both internal psychological factors such as GNIM and organizational factors such as ENTL are critical in propagating the green agenda in organizations.

One of the most critical roles that GNIM plays is in its mediation of the interrelationship between environmental leadership and sustainable employee behavior. GNIM, marked by intrinsic motivation to practice sustainability for motives beyond external influence makes an important step of psychological connection [22]. This intrinsic motivation is highly influenced by leadership that not only preaches but practices sustainability values that make eco-friendly behaviors something that is treasured on its own accord and thus develops a culture of the organization where ecofriendly behaviors become the desired way of life [33]. As proved by De Young [52], inner motivation can be a powerful tool for sustainable behavioral change, and this is evident from the basic human desire for competence in their environment. More so, the work of Ericson, Kjønstad [53] demonstrated that mindfulness and intrinsic values are instrumental in creating sustainable behaviors, in other words GNIM can have an influence on behaviors of the employees in the direction of environment protection.

Under this consideration, GNIM is revealed as a mediator by translating the perception and moral behaviors of green transformational leaders into personal behaviors and attitudes that are aligned with the organization’s sustainability goals. The inherent self-gratification arising from being part of environmental integrity leads employees to use and uphold green processes, thereby significantly increasing the chance of sustainable practices becoming embedded within the organization. Emphasis on the core, the leaders who have sound environmental conscientiousness play the driving role in introducing sustainable habits within the organizations. By the power of inspiration that they put into their words and by their actions to foster environmental responsibility among their employees, they improve GNIM, encouraging people to act more environmentally friendly. From the perspective of our underpinning theory, these leaders assume the role of example setters to persuade their teams to practice sustainability. In addition, their moves serve the basis of environmentally friendly business strategy as well as correspond with UN-SDGs, particularly those related to education, consumption and climate action. These leaders, through the cultivation of the culture of sustainability and learning, contribute significantly to organizational and environmental wellness.

**H2:** Environmentally-focused transformational leadership has a positive influence on employees’ green intrinsic motivation
**H3:** Employees’ green intrinsic motivation has a positive influence on their energy-efficient sustainable behavior
**H4:** Employees’ green intrinsic motivation mediates the positive influence of environmentally-focused transformational leadership on their energy-efficient sustainable behavior

Passion is a powerful, affirmative sentiment that moves people to conduct activities they regard significant [54]. For instance, Robertson and Barling [55] took this notion further by applying it to environmental sustainability. They coined the term ‘pro-environmental harmonious passion or GRP. It means having optimistic emotions that compel a person’s desire to participate in various sustainable programs for saving nature or life around us. As far as GRP is concerned, it goes beyond individualism and becomes an attribute of the whole society or group. If someone is passionate about the environment, he or she will naturally express passion thereby influencing others [56]. This emotional contagion process allows employees to develop shared positive emotions about environmental protection. Thus, discussion of GRP transcends personal boundaries to encompass team-wide, group-wide or organization-wide perspectives. In other words, an employee’s GRP refers to shared positive emotions causing an urge within individuals to get involved in different environmentally-driven programs [57].

The leaders’ environmental preferences help employees understand the importance of company’s green targets [58]. A transformational style of leadership influences employee values congruence and triggers positive emotions leading to optimism towards change processes [56]. To be more specific, ESTFL boosts employees’ GRP in two phases; firstly leader-member transfer followed by member-member transfer. By acting as charismatic role models for their subordinates, leaders are able to transmit their passion for the environment through idealized influence [59]. Stage two comprises of leaders transmitting environmental passion to their subordinates. Employees that have high levels of membership stability, social interdependence and expression ability are most at risk from emotional contagion. ENTL contributes to GRP which in turn increases employees’ involvement in more sustainable ways of using energy [60].

Other researchers also recognized passion as a mediating mechanism within a leadership perspective [33, 61, 62]. GRP, covering all spheres of green training, sustainable resources management, and eco-friendly practices, perform those principles and beliefs of the organization. These approaches make employees structured and properly resourced to perform environmentally conscious actions, providing a catalyst to turn organizational sustainability policies into individual practices. According to Ari, Karatepe [63], the main focus areas of green human resources management (GHRM) practices are green training and empowerment, which support the development of pro-environmental behaviors in employees. Their theoretical model explains how HR practices with GHRM can contribute to environmentally-friendly behaviors of individuals, because they make better work engagement and more satisfaction, suggesting that the sustainability-oriented actions of employees can be significantly influenced by GPR. As summed up, GRP builds the connection between organizational policies and employee behavior through providing the pertinent knowledge, skills, and motivational context for adherence to sustainable practices. With GRP, the organizations might be able to convert their environmental visions and policies into practices and behaviors that they conduct on the day to day level, helping to increase the sustainability level of their operations. Based on this stream of literature, we believe that an ENTL fosters GRP which then results into EESB.

From the standpoint of social learning theory, employees learn by observing their leaders and apply these acquired skills into practice. In essence, GRP mediates the relationship between organizational policies and employee behavior by providing the necessary knowledge, skills, and motivational context for sustainable action. Therefore:

**H5:** Environmentally-focused transformational leadership has a positive influence on employees’ green passion
**H6:** Employees’ green passion has a positive influence on their energy-efficient sustainable behavior
**H7:** Employees’ green passion mediates the positive influence of environmentally-focused transformational leadership on their energy-efficient sustainable behavior

Human values have a large impact on the behavior of employees in an organization for environmental sustainability [64, 65]. A person with GNA is one who has an intrinsic concern about the environment and a willingness to act towards improving the environmental well-being beyond self-interests [66, 67]. In relation to organizations, employees with high levels of GNA are likely to participate in EESB due to their natural concern for the environment. In this case, ENTL’s role becomes very paramount. This is because, besides practicing sustainable practices themselves, ENTL encourages his/her staff members to do so. These leaders adopt sustainable development principles and strive to align organizational goals with UN-SDGs objectives.

However, sometimes there is no straight link between ENTLs’ influence on EESB among their subordinates. It is when human values come into play especially GNA [68]. GNA serves as a buffer that strengthens relationships mediated through GNIM and GRP between ENTL and EESB. Employees who possess strong GNA have enhanced GNIM and GRP which further shapes their EESB. Because of passion towards sustainability motivation deep-seated within these individuals exists; thus compelling them towards actions beneficial to the environment. This process is guided by social learning theory. ENTL demonstrates behaviors that can be then used as examples by their subordinates. They serve as models for other employees’ observation and imitation where their practices influence them in acting in a manner that promotes sustainable culture within the company. This learned conduct when supported by the organization leads to GNIM & GRP development. Their learned behaviors become more pronounced in presence of strong GNA thereby leading to high EESB levels among these workers. Therefore,

**H8:** There exists a moderating mechanism of green altruism in the mediated paths between environmentally focused transformational leadership and energy-efficient sustainable behavior of employees through (a) green intrinsic motivation (b) green passion

Table 1 provides theoretical foundations for hypotheses

**Table 1:** Theoretical foundation with supporting reference for hypotheses.

Hypothesis	Description	Theoretical Constructs	Key References
H1	ENTL positively influences EESB.	Leaders who inspire and engage with employees on sustainability lead to improved organizational behaviors.	Bass and Riggio [69]
H2	ENTL positively influences employees’ GNIM.	Supportive environments enhance intrinsic motivation.	Deci and Ryan [25]
H3	GNIM positively influences EESB.	Intrinsically motivated behaviors are sustainable and lead to higher performance.	Ari, Karatepe [63]
H4	GNIM mediates the relationship between ENTL and EESB.	Intrinsic motivation can explain the impact of leadership on employee behavior.	Faraz, Ahmed [22]
H5	ENTL positively influences employees’ GRP.	Inspiring leadership can foster intense emotional connections to activities, such as environmental conservation.	Vallerand, Rousseau [24]
H6	GRP positively influences EESB.	Passion towards an activity leads to persistent and engaged behaviors, enhancing performance outcomes.	Jia, Liu [56]
H7	GRP mediates the relationship between ENTL and EESB.	Mediation under Passion Theory illustrates how emotional engagement, driven by leadership, impacts organizational outcomes.	Obeng, Zhu [61]
H8	Green Altruism moderates the relationships mediated by GNIM and GRP between ENTL and EESB.	Moderation suggests that personal values like altruism can strengthen or weaken the pathways from leadership through motivation and passion to behaviors.	Bautista, Dui [67] and Iyer, Davari [66]

## 3. Methodology

### 3.1 Participants and procedure

This research employed cross-sectional design using one independent variable which was ENTL while EESB served as dependent one in Chinese hospitality industry. We conducted this study specifically within Shanghai, China targeting both local and international hotels. The study targeted hotels in Shanghai, China, specifically aiming to include a representative mix of both local and international establishments to capture diverse practices and perspectives on sustainability. In total, 15 hotels participated in the survey including10 local hotels and 5 international chains, all located within the metropolitan area of Shanghai. These hotels were selected based on their engagement in sustainability initiatives and their willingness to participate in academic research. The selection process involved initial contact via email, followed by meetings to discuss the research objectives and the confidentiality of the responses. To further ensure the representativeness and reliability of the findings, the sampling methodology was carefully crafted to include hotels of various sizes and customer bases. The criteria for selecting the hotels were based on their reported sustainability practices and geographic diversity within Shanghai. This stratified approach helps in minimizing sampling bias and enhances the generalizability of the study findings across the Shanghai hospitality sector. The key participants were hotel workers who took part in our research survey. The data gathering was done in three stages to ensure completeness and precision of respondents’ replies. The process began in the beginning of March 2023, gathering demographic data and initial assessment of the ENTL. The second stage, held two weeks after, concerned the assessment of GNIM and GRP among the participants. The last stage, a two-week measurement of GNA and EESB took place. This staged approach provided us with the opportunity to limit the possibility of biases and increase response accuracy, which resulted in a final recovery rate of 60.14% after accounting for incomplete surveys.

Our study involved seven hotels which willingly agreed to be part of this data collection activity. With their participation, we were able to include a large pool of employees. Participants were chosen randomly from various departments and job roles within these hotels in order to cover diverse range of functions. In line with Helsinki Declaration, self-administered questionnaire was employed for obtaining responses anonymously ensuring that confidentiality is maintained [70, 71]. All subjects were informed on what they should expect from the study and were sought their consent before taking part in filling out surveys. We ensured them that their involvement will not extend beyond academic purposes only and by any reason they could withdraw at any time without facing any consequences.

### 3.2 Ethics statement

The observational research, "Sustainable sojourns: fostering sustainable tourism practices to meet UN-SDGs," has been ethically cleared by Shanxi Finance & Taxation College, Taiyuan, China (SFTC/Res/IRB/AL/2022/10/018). The study utilizes publicly available data, posing minimal risk, and received a waiver for a full Institutional Review Board (IRB) review. Any changes in protocol necessitating a full IRB review will be promptly communicated to the board for approval. Evidence of ethical compliance can be provided upon request.

### 3.3 Instrument, measure, and sample size

The instrument employed for data collection in this study was a self-administered questionnaire adapted from various reputable and published sources. The questionnaire was carefully designed in order to capture the multilevel dynamics of ENTL and its influence on EESB, based on well-established scales in the literature. Questions were developed under the broad spectrum of environmental sustainability behaviors and attitudes among the hospitality industry, and were used to ensure they were related to the daily operations of the participants. For example, the questions asked related to ENTL included the inquiry about leaders’ environmental practices and their roles in creating eco-friendly organizational culture. The EESB items measured energy use-related habitual activities, including compliance with energy-saving rules and active participation in sustainability activities. The questionnaire was designed with a five-point Likert scale format and divided into two main sections; the respondents’ socio-demographic characteristics and rating of items on a five-point scale. To ensure the validity and reliability of our questionnaire, we conducted a pilot study with the participation of 30 hotel employees excluded from the main study. The outcome from this phase of feedback was some minor changes for clarity and relevance. Next, the drafted questionnaire underwent a thorough validation process, which included both exploratory and confirmatory factor analyses, to determine if the scales used actually measure what they claim to measure. Cronbach’s alpha values were calculated for each scale to check internal consistency, with all coefficients exceeding the acceptable limit of 0.70, thus, indicating satisfactory reliability. The multiple correlation among the variables revealed that our hypotheses were empirically supported (detail is given in the Results section). Printed copies of questionnaires were given to each respondent during the study for convenience and accuracy in response.

The measures used in this study to evaluate different variables were borrowed from previous researches. ENTL, as an independent variable, has been measured using 12 items adapted from Robertson [72] on employees’ perceptions of leader behaviors such as inspirational motivation, intellectual stimulation, individualized consideration, and environmental influence. The criterion variable EESB was assessed by 8-items borrowed from Blok, Wesselink [73], which specifically addressed electricity related behaviors like lighting, heating or cooling and use of computer at office. In addition GNIM (6-items) and GRP (10-items) are intervening variables measured through scales taken from by Li, Bhutto [41] and Robertson and Barling [55], respectively. Finally GNA was measured using four items scale developed by Schwartz [74].

The minimum sample size determination relied on A-priori sample size calculator which is ideal for structural equation modeling [75–77]. Based on six latent variables with 40 observed variables it suggested that a minimum sample size would be around 400. In order to ensure a higher number of responses, we distributed 700 surveys among hotel employees. We received 451 filled questionnaires. However, some were excluded due to incomplete information. After the data cleaning process, 420 responses were incorporated into the final dataset for analysis.

In terms of the demographic composition of our sample, both genders were represented, though male respondents dominated slightly, comprising 58% of the total. A significant portion of the respondents, approximately 78%, possessed work experience ranging from 4 to 10 years. As for age, the majority fell within the 18–55 years bracket, accounting for 89% of the total sample.

## 4. Results

### 4.1 Initial analysis

Table 2 presents the factor analysis results that were utilized to examine the outer factor loadings as well as test the convergent validity of measurement model. The table has five constructs namely EESB, ENTL, GNA, GNIM and GRP which are made up of several items each. Upon reviewing these factor loadings closely, it can be seen that most items have factor loadings exceeding the acceptable threshold of 0.70 [78, 79] meaning strong loading on their respective factors. However, for purposes of improving this model’s quality as well as strengthening its convergence validity; four items were dropped out from it due to low factor loading with others. T-values show that almost all item values are highly significant indicating high degree relationships amongst variables involved in this study.

**Table 2:** Confirmatory factor analysis.

	Items	OS	M	S.D	T
	EESB2 <- EESB	0.818	0.819	0.027	29.773
	EESB3 <- EESB	0.756	0.757	0.039	19.444
	EESB4 <- EESB	0.765	0.767	0.061	12.559
**EESB**	EESB5 <- EESB	0.706	0.709	0.067	10.577
**AVE = 0.636**	EESB6 <- EESB	0.846	0.846	0.023	37.097
	EESB7 <- EESB	0.835	0.836	0.031	27.256
	EESB8 <- EESB	0.844	0.844	0.021	39.426
	ENTL1 <- ENTL	0.865	0.864	0.019	46.606
	ENTL10 <- ENTL	0.878	0.876	0.018	47.891
	ENTL11 <- ENTL	0.849	0.848	0.022	39.092
**ENTL**	ENTL12 <- ENTL	0.868	0.867	0.02	42.882
**AVE = 0.651**	ENTL3 <- ENTL	0.809	0.809	0.031	25.712
	ENTL5 <- ENTL	0.641	0.64	0.062	10.338
	ENTL6 <- ENTL	0.808	0.806	0.032	25.455
	ENTL7 <- ENTL	0.626	0.625	0.053	11.823
	ENTL8 <- ENTL	0.811	0.811	0.029	28.238
	ENTL9 <- ENTL	0.863	0.863	0.021	41.389
	GNA1 <- GNA_	0.826	0.825	0.026	31.771
	GNA2 <- GNA_	0.813	0.813	0.03	26.861
**GNA**	GNA3 <- GNA_	0.83	0.83	0.025	33.076
**AVE = 0.694**	GNA4 <- GNA_	0.863	0.863	0.022	38.418
	GNIM1 <- GNIM	0.750	0.751	0.033	22.995
	GNIM2 <- GNIM	0.738	0.74	0.051	14.577
	GNIM3 <- GNIM	0.836	0.836	0.024	34.966
**GNIM**	GNIM4 <- GNIM	0.853	0.853	0.021	40.853
**AVE = 0.597**	GNIM5 <- GNIM	0.821	0.82	0.029	28.343
	GNIM6 <- GNIM	0.611	0.609	0.046	13.219
	GRP1 <- GRP	0.667	0.667	0.048	13.852
	GRP10 <- GRP	0.761	0.76	0.034	22.426
	GRP2 <- GRP	0.684	0.684	0.037	18.422
**GRP**	GRP5 <- GRP	0.778	0.778	0.03	25.602
**AVE = 0.544**	GRP6 <- GRP	0.773	0.776	0.046	16.969
	GRP7 <- GRP	0.749	0.749	0.039	19.254
	GRP8 <- GRP	0.746	0.746	0.033	22.764
	GRP9 <- GRP	0.736	0.735	0.057	12.875

**Notes:** λ = Item loadings, AVE = Average variance extracted, OS = Original sample, M = mean sample, T = *t*-values

**Fig 1:**
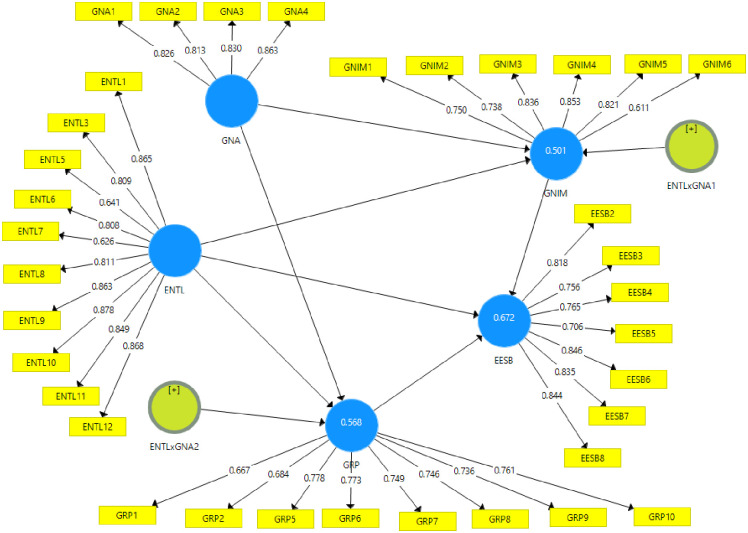
The measurement model.

Another confirmation for convergent validity is the analysis of the average variance extracted (AVE). In this study, AVE values are: 0.636 for EESB; 0.651 for ENTL; 0.694 for GNA; 0.597 for GNIM and GRP of 0.544. Generally, if AVE values exceed 0.50, that indicates it can be satisfactory indicating that, on average, the construct explains more than half of the variance of its items [80, 81]. All constructs in this research meet this requirement but GNIM and GRP have figures that are relatively closer to the required limit. From these findings, it can be concluded that measurement model has a satisfactory convergent validity. The measurement model is presented in Fig 1.

In Table 3, Cronbach’s alpha, rho_A and composite reliability which are three widely accepted measures of reliability are given. For EESB Cronbach alpha is at 0.904, rho_A at 0.906 while composite reliability stands at 0.924. The ENTL construct exhibits even higher reliability, with Cronbach’s alpha at 0.938, rho_A at 0.943, and composite reliability at 0.948. GNA’s Cronbach alpha is at 0.853, rho_A being at 0.855 and composite reliability standing at 0.901. GNIM reports a Cronbach alpha value of 0.862, rho_A = 0.872 and composite reliability = 0.898. Finally, GRP shows a Cronbach alpha and rho_A = 0.880 while composite reliability equals to 0.905. All these statistics across all three measures indicate high internal consistency and therefore high levels of reliability as they exceed traditional threshold (0.70). This suggests that these constructs have strong internal cohesiveness among the items and are truly reflective of the common underlying latent variables.

**Table 3:** Reliability statistics.

Variable	Cronbach alpha	Rho_A	Composite reliability
**EESB**	0.904	0.906	0.924
**ENTL**	0.938	0.943	0.948
**GNA**	0.853	0.855	0.901
**GNIM**	0.862	0.872	0.898
**GRP**	0.88	0.88	0.905

The following major insights can be gleaned from Table 4 which deals with discriminant validity and inter-construct correlations. The diagonal entries represent square root of AVE for each construct which should ideally be larger than corresponding off-diagonal elements in the respective rows and columns for adequate discriminant validity (0.797, EESB; 0.807, ENTL; 0.833, GNA; 0.773, GNIM and GRP = 0.738). The EESB construct exhibits moderate to strong correlation with other constructs as shown by values ranging from 0.443 to 0.668. Similar pattern is noticed in ENTL build where there are correlation values within a range of 0.599 to 0.678. The correlations between GNA and other variables are also distinguishable since they vary from 0.443 to 0.645. GNIM and GRP have a correlation of 0.673. This implies that each construct captures variance distinct from the others, validating the individuality and relevance of each construct in the study. Also, significant associations between factors attest to an interconnected nature of variables under investigation.

**Table 4:** Correlations and discriminant validity.

	**EESB**	**ENTL**	**GNA**	**GNIM**	**GRP**
**EESB**	0.797				
**ENTL**	0.629	0.807			
**GNA**	0.443	0.599	0.833		
**GNIM**	0.668	0.664	0.563	0.773	
**GRP**	0.773	0.678	0.645	0.673	0.738

Table 5 presents the Heterotrait-Monotrait Ratio (HTMT) values which indicate whether there is a discriminant validity among five constructs: EESB, ENTL, GNA, GNIM and GRP. Ideally, HTMT values should be below 0.90 or in some cases below 0.85 for adequate discriminant validity. The value of HTMT between EESB and ENTL is 0.684 while that between EESB and GNA stands at 0.502; both are way below the threshold of 0.850 suggesting good discriminant validity between these two variables. GNIM and EESB’s HTMT value is also less than even the regular value to show there are adequate sign of differences between them. For ENTL and GNA, their HTMT value was found at 0.668 representing a case which it can be assumed that they have different features from one another. Also, GNIM has got lower scores when considered separately with ENTL and again with GNA standing at 0.734 and 0.659 respectively: both cases do not exceed the threshold of 0.850. GRP’s HTMT values with respect to EESB, ENTL, GNA, and GNIM are 0.655, 0.741, 0.747, and 0.798, respectively. These values are below the conventional threshold, indicating discriminant validity.

**Table 5:** Hetrorait-Monotrait Ratio (HTMT).

	**EESB**	**ENTL**	**GNA**	**GNIM**	**GRP**
**EESB**					
**ENTL**	0.684				
**GNA**	0.502	0.668			
**GNIM**	0.766	0.734	0.659		
**GRP**	0.655	0.741	0.747	0.798	

### 4.2 Structural analysis

**Fig 2:**
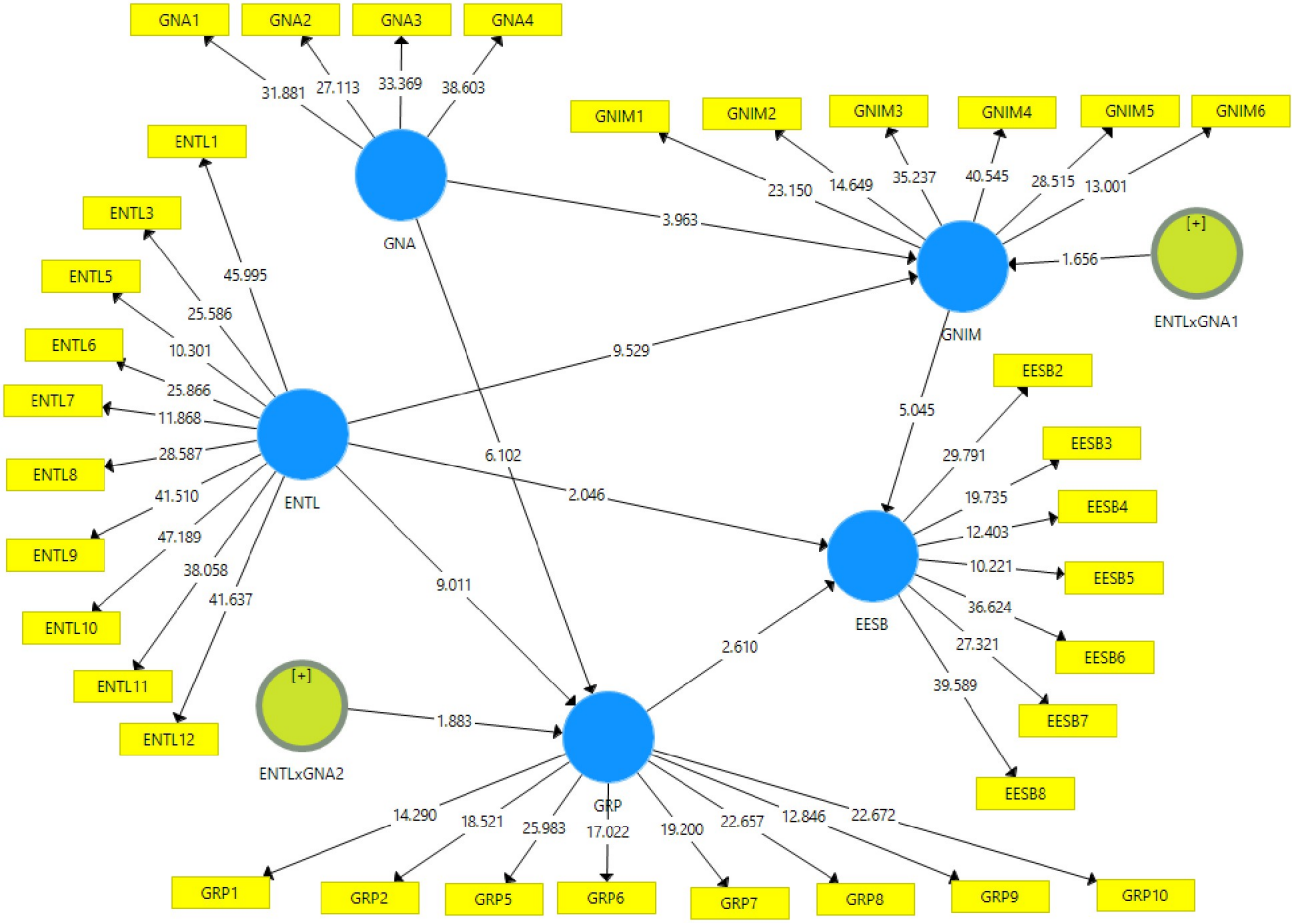
The structural model.

For structural analysis, we used SMART-PLS software which is a contemporary data analysis tool, known for its capability and flexibility in handling with complex models [80, 82]. The structural analysis (Table 6) indicates significant associations among constructs via direct effects, mediation, and moderated mediation. ENTL’s impact on EESB is significant (β = 0.129, p = 0.038, intervals = 0.004–0.251), and its influence on GNIM and GRP is highly substantial (β = 0.553 and 0.5, p≈0, intervals = 0.432–0.660 and 0.388–0.601 respectively). The paths from GNIM to EESB (β = 0.484, p≈0) and GRP to EESB (β = 0.263, p = 0.009) are also significant, corroborating hypotheses H1, H2, H3, H5, and H6. In the mediation analysis, GNIM and GRP significantly bridge ENTL’s influence on EESB (β = 0.268 and 0.132, p≈0 and 0.016, intervals = 0.154–0.405 and non-zero, respectively). The moderated mediation paths, ENTLxGNA1 to GNIM to EESB (β = 0.099, p = 0.033, intervals = 0.054–0.205) and ENTLxGNA2 to GRP to EESB (β = 0.124, p = 0.020, intervals = 0.028–0.244), exhibit marginal significance, highlighting intricate relationships. This substantiates hypotheses H4, H7, and H8. Fig 2 summarizes the structural model.

**Table 6:** Hypotheses analysis.

	Original Sample (O)	Sample Mean (M)	Standard Deviation (STDEV)	T Statistics (O/STDEV)	P Values	LLCI 2.50%	ULCI 97.50%
**ENTL -> EESB**	0.129	0.125	0.062	2.072	0.038	0.004	0.251
**ENTL -> GNIM**	0.553	0.558	0.058	9.603	0.000	0.432	0.66
**ENTL -> GRP**	0.5	0.505	0.055	9.081	0.000	0.388	0.601
**GNIM -> EESB**	0.484	0.488	0.095	5.106	0.000	0.297	0.67
**GRP -> EESB**	0.263	0.265	0.101	2.613	0.009	0.058	0.454
**ENTL -> GNIM -> EESB**	0.268	0.273	0.064	4.168	0.000	0.154	0.405
**ENTLxGNA1 -> GNIM -> EESB**	0.099	0.036	0.042	2.750	0.033	0.054	0.205
**ENTL -> GRP -> EESB**	0.132	0.135	0.055	2.411	0.016	0.062	0.21
**ENTLxGNA2 -> GRP -> EESB**	0.124	0.053	0.023	2.339	0.020	0.028	0.244

**Notes:** CI = 95% confidence interval with lower and upper limits, O-sample = original sample, M-sample = mean sample

## 5. Discussion

This study aimed to examine the relationship between ENTL and EESB using the social learning theory as a theoretical lens. Theoretical predictions (H1, H2, H3, and H4) were found statistically significant, substantiating the key role that ENTL plays in promoting GNIM and consequential EESB. Our results indicate that transformational leadership with an environmental focus can lead to employees’ energy-efficient sustainable behavior changes (H1). This supports the social learning theory argument of people imitating other people’s actions especially those by influential persons such as leaders. By so doing, they serve as examples for their subordinates who will also improve on their environment-friendly manner of performing activities thereby leading to better EESB.

In addition, this study discovered that GNIM is critical in encouraging EESB (H3). Social learning theory suggests that behavioral change begins once behaviors and attitudes demonstrated by leaders are internalized by followers. In line with these findings of previous scholars like Deci and Ryan [42] state that when employees are intrinsically motivated towards preserving the ecological system it is reflected in their energy-specific behavior. Furthermore, our research established a positive association between ENTL and employees’ GNIM (H2), which mediates the positive influence of ENTL on EESB (H4). This means that transformational leaders who value environmental conservation are not only role models but also catalysts for a psychological process through which workers get internally motivated to be environmentally conscious.

The results have important implications particularly for China which has major environmental challenges amid its quest for rapid economic growth. As China undergoes industrialization at a breakneck speed coupled with urbanization; it is necessary to create a culture of environmental accountability among corporate entities so as to strike a balance between economic growth and environmental protection. To achieve this equilibrium, organizations need to embrace ecologically driven transformational leadership approaches that affect GNIM amongst the workforce and energy-saving behaviors. At the same time, Chinese organizations can make use of ENTL as a teaching tool in order to bring behavioral change among their own employees. Based on this study, it is clear that with China’s commitment to some of the UN-SDGs such as clean and sustainable energy use (SDG 7), responsible consumption and production patterns (SDG 12) and mitigating climate change (SDG 13), ENTL could be used for achieving these objectives.

This is also supported by our findings for H5, H6, H7, and H8. In this regard, the results of our research show that ENTL has a positive impact on the GRP of employees (H5) according to the precepts of social learning theory in which behaviors and values are learned through observation as indicated by Bandura and McClelland [30]. We further conclude from this study that the Chinese hospitality organizations need to develop leaders who can act as role models for environmental sustainability. Apparently, there is a chain reaction between these kinds of leaders’ practices and their organizations that inspire their employees to behave in likewise and highly motivate them towards environmental conservation. This is in line with Robertson and Barling [55]. The other finding from our research was an increase in the GRP, means an increase in EESB (H6); some earlier studies have discussed how passionate motivation leads individuals to engage in activities they perceive as important [54]. This holds tremendous significance for Chinese organizations looking to foster a culture of sustainability. Due to encouraging a desire for employees’ participation in sustainability initiatives by GRP [57], leaders should aim at creating such enthusiasm among their teams. Additionally, we discovered that GRP mediated the positive effect of ENTL on EESB within employees (H7). As explained by previous researches considering passion’s mediating function within leadership contexts [33, 61, 62]. This crucial insight indicates that the infusion of passion into an employee’s experience could catalyze the translation of sustainable leadership values into tangible energy-efficient behaviors.

However, one of the most interesting things concerning our current investigation is GNA as a moderator between ENTL-EESB path through GNIM and GRP mediation (H8). According to this observation if workers have strong GNA then their GNIM and GRP will be increased thus positively affecting their EESB [66, 67]. Understanding how personal values affect organizational behavior provides a more sophisticated perspective on the pathway toward environmental sustainability. Our study supports these hypotheses and shows the way in which sustainable cultures are nurtured within organizations. Moreover, this research demonstrates that social learning theory is relevant to understanding the role of transformational leadership in promoting environmental sustainability, which is highly significant for rapidly developing economies like China. Due to rapid economic development in the country there has been a need for parallel focus on environment conservation which renders results of such studies very valuable. Additionally, it tells us how GRP and GNA can be leveraged by firms to promote EESB. In order to ensure energy efficiency as well as embrace sustainability, Chinese companies may adopt ENTL principles; encourage GRP while fostering GNA among employees.

### 5.1 Theoretical insights

The present study makes a number of theoretical implications that expand and deepen our comprehension of ENTL, GNIM, GRP and GNA in the context of EESB in the Chinese tourism and hospitality industry. To start with, this research brings about the theoretical understanding that ENTL plays in influencing employees’ EESB. Previous works dwelled on transformational leadership on sustainable behavior but it focused on environmentally-focused transformational leadership. This focus enhances this literature immeasurably by showing how leaders can enhance sustainable behavior among their workers if they adopt an environmental perspective. Additionally, the research integrates personal components like GNIM, GRP and GNA into one model thus expanding upon existing theories as well as providing new ones. Thus, there is an opportunity to examine how individual level variables mediate between ENTL and EESB thereby creating a better understanding of the dynamics at work. In addition, by doing so it bridges this gap because there is no existing research that has shown mediation effect of these variables on sustainable behaviors.

Moreover, this investigation is unique in its in-depth exploration into GNA’s role in relation to EESB especially within hospitality business sector. Despite its potential for use underexplored GNA value has left knowledge gap regarding its impact on managing sustainability practice. Our study fills this gap revealing how important GNA can be in promoting sustainability initiatives among organizations operating in tourism and hospitality sector. Furthermore from a geographical standpoint our study also carries immense importance as it explores China’s journey towards sustainability as a rapidly developing country. Most studies conducted about developed countries resulting to lack of insights concerning specific contexts and challenges associated with emerging markets such as China; therefore our study addresses this anomaly by increasing the scope of literature geographically, so that we have a global inclusive framework.

Finally, this study is particularly salient given its focus on China’s booming tourism and hospitality sector. The rapid growth of the sector has presented unique challenges and opportunities for sustainability practices, as evidenced by visitors congregating to attractions in cities such as Shanghai, Suzhou, and Hangzhou. This study thus offers new theoretical inputs that are relevant to China’s current socioeconomic setting by looking at EESB within the industry. In summary this study is valuable because it extends theories on ENTL and its relationship with EESB through revealing the mediating role of GNIM, GRP and moderating role of GNA and examining them in the context of Chinese tourism and hospitality industry.

### 5.2 Practical insights

The findings of this research have far-reaching applications in practice for different stakeholders, with particular significance for China’s tourism and hospitality industry. These stakeholders can harness the interplay of ENTL, GNIM, GRP, and GNA to encourage EESB within their establishments. First and foremost, realizing the central importance of ENTL in nurturing EESB, it is essential for leadership training initiatives to incorporate an ecological perspective within the transformational leadership framework. Through training, organizational leaders can be equipped to stimulate and steer their workforce towards sustainable conduct and embed environmental considerations into decision-making. This method not only heightens staff involvement in green initiatives but also nurtures a corporate culture that cherishes environmental guardianship. Moreover, appreciating the intermediary roles of GNIM, GRP, and GNA in steering EESB, it is vital for organizations to create a milieu that encourages these elements. By putting into place policies and rewards that augment GNIM, like acknowledging and incentivizing employees to adopt energy conservation measures or initiating eco-friendly projects, organizations can foster GNIM. To foster GRP, organizations could create avenues or assignments that let employees channel their zeal for environmental causes. Additionally, to bolster GNA, organizations should highlight the long-lasting impact of their green efforts on society at large and future generations, thus fortifying employees’ benevolent inclinations towards EESB.

In light of the tourism and hospitality domain in China, these applications in practice are especially vital. With the industry’s swift expansion and possible ecological repercussions, stakeholders must employ ENTL to direct EESB, backed by the intermediary functions of GNIM, GRP, and GNA. For example, hotels can adopt energy-saving measures in their day-to-day operations, such as utilizing energy-efficient lighting or advocating minimal water consumption. Similarly, tourist sites can take up eco-friendly measures such as waste management and recycling schemes and communicate their dedication to environmental preservation to visitors.

Moreover, considering the allure of Chinese metropolises like Shanghai, Suzhou, and Hangzhou for travelers, it is essential for tourism authorities in these cities to endorse eco-friendly tourism practices, emphasizing the criticality of environmental preservation to both enterprises and tourists. This could encompass designing instructional programs or consciousness-raising campaigns concerning the significance of sustainable tourism and offers guidelines for local enterprises on the incorporation of sustainable measures. Similarly, the scope of these applications transcends organizational boundaries to encompass policymakers and governmental bodies. Through comprehending the dynamics revealed in this research, they can formulate policies or projects that bolster ENTL and encourage GNIM, GRP, and GNA, hence propelling EESB at a more extensive scale.

Lastly these practical insights are equally important from the perspective of UN-SDGs, especially SDG-7,9, 12 and 13. For example, a primary practical implication pertains to SDG 7 (affordable and clean energy), where fostering EESB within organizations, especially those in energy-intensive sectors like tourism and hospitality, directly contributes to improved energy efficiency. Leaders should be trained to prioritize and inspire energy-efficient practices, such as implementing energy-saving lighting or other energy-efficient technologies. Similarly, the emphasis on GNIM, GRP, and GNA supports the achievement of SDG 12 (responsible consumption and production). By fostering a culture that encourages these values, organizations can significantly reduce their environmental footprints. This can be achieved through various initiatives like setting up recycling programs, waste reduction campaigns, and incentivizing employees to take part in these initiatives. Moreover, the research’s outcomes also have significant practical implications for SDG 9 (industry, innovation, and infrastructure). Organizations can leverage the dynamics of ENTL, GNIM, GRP, and GNA to inspire innovation in developing greener infrastructures. This may involve investing in renewable energy sources or innovative technologies that reduce carbon emissions. The findings of this study also align with SDG 13 (climate action). Organizations can harness the power of ENTL to steer the direction of climate action within their operations. This includes developing climate change mitigation strategies and promoting employee participation in climate action initiatives.

### 5.3 Limitations and future prospects

This research however has a few limitations which in turn open up areas for further research. At first, it investigated associations between ENTL GNIM, GRP, GNA and EESB but only within the confines of China’s tourism and hospitality industry. As such, these findings may not be applicable to different industries or cultural and socio-economic contexts. Prospective studies can examine these relationships across various industries and locations thus making them universally valid. Further, the study used cross-sectional data as its foundation which restricts the ability to make causal inferences. Future enquiries could adopt longitudinal perspectives that would help trace these relationships over a long period thereby establishing causality. Thirdly, it looked into the facilitating roles played by GNIM, GRP, and GNA. However, other hidden mediators like ecological self-concept or organization support for pro-environmental behaviors worth exploring in future works. In order to have a full understanding of what influences EESB this should be investigated in future studies. Fourthly, while this study majorly focused on positive aspects of ENTL GNIM GRP AND GNA; there is value in checking any possible negative impacts in upcoming studies such as stress from high levels of GNIM or challenges faced when implementing EESB where resources are low. On a further note, this research relied on self-reported measures to assess GNIM, GRP GNA AND EESB that could be influenced by social desirability bias (the tendency to present ourselves favorably). Subsequent studies may consider using objective metrics or assessments administered by independent bodies to counteract these biases. Our study’s insights, based on the Shanghai hotel industry, may not directly apply to the broader tourism sector or other regions, given its specific focus. Generalizing these findings to different tourism contexts should be done with caution. The need for further research across diverse tourism sectors and locations is evident to broaden our understanding of sustainable practices and their contribution to achieving the UN-SDGs globally. Lastly, while our findings contribute valuable insights into the role of leadership and motivational factors in promoting sustainability, the research did not extend to analyzing the impact of demographic factors on EESB. The exclusion of a detailed demographic analysis for example including variables such as age, gender, educational background, and years of service may constitute a limitation of this study which can be addressed in future studies.

### 5.4 Conclusion

To sum it up, through this work we have made significant steps towards filling the theoretical gaps regarding environmental sustainability especially with respect to personal factors like GNIM, GRP, and GNA on sustainable behavior within organizations concerned with environmental issues. This also established how transformational leadership focuses on environment that could be used to drive a sustainable behavior of the employees in view of China’s growing tourism and hospitality sector. The investigation shows that ENTL is related with these individual factors in order to promote EESB which can mitigate global climate change and help support UN-SDGs. In today’s world where everyone deals with climate matters, this study’s findings will help in ensuring sustainability in organizations especially the ones on high operation sectors like tourism and hospitality. Through highlighting the importance of ENTL, as well as personal factors like GNIM, GRP and GNA towards promoting EESB, it serves as an inspiration to organizations particularly within the emerging tourism and hospitality industry in China to adopt sustainable practices and nurture eco-friendly culture around them. With this, such firms stand a chance of enjoying substantial economic benefits while at the same time creating a better life for people, creating more wealth and contributing significantly to efforts aimed at attaining carbon neutrality.

### 5.5 Specific recommendations

Based on the findings of this study, several recommendations can be put forward for tourism and hospitality organizations, particularly in the Chinese context, which would help in facilitating the UN-SDGs. We, in this regard, propose the following recommendations:

- Promote ENTL: One way to achieve this is through training and skill development in ecologically conscious decision making; setting clear benchmarks for sustainability, and acting as models of sustainable behavior. This would enhance GNIM among employees, contributing to SDG 12 and SDG 13.
- Cultivate GNIM and GRP: Organizations should endeavor to create a culture that appreciates environment-friendly practices. Organizations could go about this by rewarding workers who are energy efficient with prizes, organizing environmental activities as team building activities, or providing learning opportunities on the subject matter. By doing so, they will help nurture GNIM plus GRP which again fits into SDG 4.
- Encourage GNA: Additionally, organizations can encourage green altruism through engaging in corporate social responsibility initiatives that safeguard the environment; involvement in community green programs; and promoting eco-friendly habits among the customers as well as suppliers. This could help foster an organizational culture that values sustainability and contribute to SDGs like SDG 15 (life on land) and SDG 14 (life below water).
- Prioritize energy-efficient practices: It is therefore essential that tourism and hospitality businesses prioritize their energy usage. For instance, they may opt for appliances designed to conserve energy instead of those which do not save energy at all. In order to reduce power wastage areas where much electricity is spent may be identified through regular scrutinizes of the hotel premises. Achieving these objectives therefore supports SDG’s 7 & 9.
- Adopt sustainable business models: Henceforth, it can employ green business strategies such as producing products that are environmentally friendly or services along a similar line, including competition enhancing value propositions founded upon ecological stewardship to align with SDG 12.

## Supporting information

S1 Data (TXT)

## References

[1] Kong L, Sial MS, Ahmad N, Sehleanu M, Li Z, Zia-Ud-Din M, et al. CSR as a potential motivator to shape employees’ view towards nature for a sustainable workplace environment. Sustainability. 2021;13(3):1499.

[2] Ahmad N, Samad S, Mahmood S. Sustainable pathways: the intersection of CSR, hospitality and the United Nations’ sustainable development goals. Current Issues in Tourism. 2024:1–20.

[3] Yu H, Shabbir MS, Ahmad N, Ariza-Montes A, Vega-Muñoz A, Han H, et al. A contemporary issue of micro-foundation of CSR, employee pro-environmental behavior, and environmental performance toward energy saving, carbon emission reduction, and recycling. International Journal of Environmental Research and Public Health. 2021;18(10):5380. doi: 10.3390/ijerph18105380 34070088 PMC8158375

[4] Liu C, Ahmad N, Jiang M, Arshad MZ. Steering the path to safer food: The role of transformational leadership in food services to combat against foodborne illness. Journal of Retailing and Consumer Services. 2024;81(Ahead of print):103958.

[5] Filimonau V, Santa Rosa M, Franca LS, Creus AC, Ribeiro GM, Molnarova J, et al. Environmental and carbon footprint of tourist accommodation: A comparative study of popular hotel categories in Brazil and Peru. Journal of cleaner production. 2021;328:129561.

[6] Dubois G, Sovacool B, Aall C, Nilsson M, Barbier C, Herrmann A, et al. It starts at home? Climate policies targeting household consumption and behavioral decisions are key to low-carbon futures. Energy Research & Social Science. 2019;52:144–58.

[7] UNEP. Emissions Gap Report 2022: United Nations Environmental Program; 2022 [February, 02, 2023]. https://www.unep.org/resources/emissions-gap-report-2022?gclid=Cj0KCQjwsIejBhDOARIsANYqkD37riJrQoRJI91Vjq23kuJKCRJRulntu_tr2XQqmQ_MVeR4GDRpFOIaArKIEALw_wcB.

[8] Ahmad N, Ullah Z, Arshad MZ, waqas Kamran H, Scholz M, Han H. Relationship between corporate social responsibility at the micro-level and environmental performance: The mediating role of employee pro-environmental behavior and the moderating role of gender. Sustainable Production and Consumption. 2021;27:1138–48.

[9] Murtaza SA, Mahmood A, Saleem S, Ahmad N, Sharif MS, Molnár E. Proposing stewardship theory as an alternate to explain the relationship between CSR and Employees’ pro-environmental behavior. Sustainability. 2021;13(15):8558.

[10] Guan X, Ahmad N, Sial MS, Cherian J, Han H. CSR and organizational performance: The role of pro‐environmental behavior and personal values. Corporate Social Responsibility and Environmental Management. 2023;30(2):677–94.

[11] Ahmad N, Scholz M, AlDhaen E, Ullah Z, Scholz P. Improving firm’s economic and environmental performance through the sustainable and innovative environment: evidence from an emerging economy. Frontiers in Psychology. 2021;12:651394. doi: 10.3389/fpsyg.2021.651394 34803789 PMC8599963

[12] Chaudhary R. Green human resource management and employee green behavior: an empirical analysis. Corporate Social Responsibility and Environmental Management. 2020;27(2):630–41.

[13] Ahmad N, Ahmad A, Siddique I. Responsible Tourism and Hospitality: The Intersection of Altruistic Values, Human Emotions, and Corporate Social Responsibility. Administrative Sciences. 2023;13(4):105.

[14] Ahmad N, Ahmad A, Lewandowska A, Han H. From screen to service: how corporate social responsibility messages on social media shape hotel consumer advocacy. Journal of Hospitality Marketing & Management. 2023:1–30.

[15] Ahmad N, Ullah Z, Ryu HB, Ariza-Montes A, Han H. From corporate social responsibility to employee well-being: Navigating the pathway to sustainable healthcare. Psychology Research and Behavior Management. 2023:1079–95. doi: 10.2147/PRBM.S398586 37041962 PMC10083008

[16] Ahmad N, Ullah Z, AlDhaen E, Siddique I. Promoting the advocacy behavior of customers through corporate social responsibility: The role of brand admiration. Business and Society Review. 2023;128(2):367–86.

[17] Deng Y, Cherian J, Ahmad N, Scholz M, Samad S. Conceptualizing the role of target-specific environmental transformational leadership between corporate social responsibility and pro-environmental behaviors of hospital employees. International Journal of Environmental Research and Public Health. 2022;19(6):3565. doi: 10.3390/ijerph19063565 35329253 PMC8955964

[18] World Travel Awards. World’s Leading Tourist Board 2023 United Kingdom: World Travel Awards; 2023 [April 20, 2024]. https://www.worldtravelawards.com/award-worlds-leading-tourist-board-2023.

[19] Farrukh M, Ansari N, Raza A, Wu Y, Wang H. Fostering employee’s pro-environmental behavior through green transformational leadership, green human resource management and environmental knowledge. Technological Forecasting and Social Change. 2022;179:121643.

[20] Siemens. Siemens Energy halves emissions in its operations Berlin: Siemens.com; 2022 [Feb 09, 2024]. https://www.siemens-energy.com/global/en/home/press-releases/siemens-energy-halves-emissions-in-its-operations.html#:~:text=By%202030%2C%20Siemens%20Energy%20aims,The%20planned%20target%20was%2084%25.

[21] Kimpton. Environment: OUR PLANET, OUR RESPONSIBILITY San Francisco, California, United States: Kimpton.com; 2024 [Feb 09, 2024]. https://www.ihg.com/kimptonhotels/content/us/en/about-us/kimpton-cares/environment.

[22] Faraz NA, Ahmed F, Ying M, Mehmood SA. The interplay of green servant leadership, self‐efficacy, and intrinsic motivation in predicting employees’ pro‐environmental behavior. Corporate Social Responsibility and Environmental Management. 2021;28(4):1171–84.

[23] Cho M, Yoo JJ-E. Customer pressure and restaurant employee green creative behavior: serial mediation effects of restaurant ethical standards and employee green passion. International Journal of Contemporary Hospitality Management. 2021;33(12):4505–25.

[24] Vallerand RJ, Rousseau FL, Grouzet FM, Dumais A, Grenier S, Blanchard CM. Passion in sport: A look at determinants and affective experiences. Journal of Sport and Exercise Psychology. 2006;28(4):454–78.

[25] Deci EL, Ryan RM. The" what" and" why" of goal pursuits: Human needs and the self-determination of behavior. Psychological inquiry. 2000;11(4):227–68.

[26] Asian Development Bank. Energy in Asia and the Pacific Mandaluyong, Philippines: ADB; 2021 [March 26, 2023]. https://www.adb.org/what-we-do/topics/energy#:~:text=Source%3A%20Our%20World%20in%20Data,comes%20from%20electricity%20and%20heat.

[27] BP International. Statistical Review of World Energy Houston, USA: BP.com; 2021 [March 26, 2023]. https://www.bp.com/content/dam/bp/business-sites/en/global/corporate/pdfs/energy-economics/statistical-review/bp-stats-review-2021-full-report.pdf.

[28] Pan J. Lowering the carbon emissions peak and accelerating the transition towards net zero carbon. Chinese Journal of Urban and Environmental Studies. 2021;9(03):2150013.

[29] Por KL. China Pledges to Become Carbon Neutral by 2060 New York, USA: Global Citizen; 2020 [March 26, 2023]. https://www.globalcitizen.org/es/content/china-pledges-to-become-carbon-neutral-by-2060/?gclid=EAIaIQobChMIppeRnIm9_wIVSQmLCh28cQ2hEAAYAyAAEgJNtvD_BwE.

[30] Bandura A, McClelland DC. Social learning theory: Englewood cliffs Prentice Hall; 1977.

[31] Molnár E, Mahmood A, Ahmad N, Ikram A, Murtaza SA. The interplay between corporate social responsibility at employee level, ethical leadership, quality of work life and employee pro-environmental behavior: the case of healthcare organizations. International Journal of Environmental Research and Public Health. 2021;18(9):4521. doi: 10.3390/ijerph18094521 33923201 PMC8123181

[32] Althnayan S, Alarifi A, Bajaba S, Alsabban A. Linking environmental transformational leadership, environmental organizational citizenship behavior, and organizational sustainability performance: A moderated mediation model. Sustainability. 2022;14(14):8779.

[33] Li Z, Xue J, Li R, Chen H, Wang T. Environmentally specific transformational leadership and employee’s pro-environmental behavior: The mediating roles of environmental passion and autonomous motivation. Frontiers in psychology. 2020;11:1408. doi: 10.3389/fpsyg.2020.01408 32670165 PMC7330121

[34] Peng J, Samad S, Comite U, Ahmad N, Han H, Ariza-Montes A, et al. Environmentally specific servant leadership and employees’ Energy-specific pro-environmental behavior: Evidence from healthcare sector of a developing economy. International Journal of Environmental Research and Public Health. 2022;19(13):7641. doi: 10.3390/ijerph19137641 35805297 PMC9266249

[35] Xu L, Mohammad SJ, Nawaz N, Samad S, Ahmad N, Comite U. The role of CSR for de-carbonization of hospitality sector through employees: A leadership perspective. Sustainability. 2022;14(9):5365.

[36] Mi L, Gan X, Xu T, Long R, Qiao L, Zhu H. A new perspective to promote organizational citizenship behaviour for the environment: The role of transformational leadership. Journal of Cleaner Production. 2019;239:118002.

[37] Singh SK, Del Giudice M, Chierici R, Graziano D. Green innovation and environmental performance: The role of green transformational leadership and green human resource management. Technological forecasting and social change. 2020;150:119762.

[38] Robertson JL, Barling J. Contrasting the nature and effects of environmentally specific and general transformational leadership. Leadership & Organization Development Journal. 2017;38(1):22–41.

[39] Rizvi YS, Garg R. The simultaneous effect of green ability-motivation-opportunity and transformational leadership in environment management: the mediating role of green culture. Benchmarking: An International Journal. 2021;28(3):830–56.

[40] Khan AN, Khan NA. The nexuses between transformational leadership and employee green organisational citizenship behaviour: Role of environmental attitude and green dedication. Business Strategy and the Environment. 2022;31(3):921–33.

[41] Li W, Bhutto TA, Xuhui W, Maitlo Q, Zafar AU, Bhutto NA. Unlocking employees’ green creativity: The effects of green transformational leadership, green intrinsic, and extrinsic motivation. Journal of cleaner production. 2020;255:120229.

[42] Deci EL, Ryan RM. Intrinsic motivation and self-determination in human behavior: Springer Science & Business Media; 2013.

[43] Saif N, Goh GGG, Ong JW, Khan IU. Green transformational and transactional leadership in fostering green creativity among university students. Global Journal of Environmental Science and Management. 2023;9(3):577–88.

[44] Wu J, Chen D, Bian Z, Shen T, Zhang W, Cai W. How does green training boost employee green creativity? A sequential mediation process model. Frontiers in Psychology. 2021;12:759548. doi: 10.3389/fpsyg.2021.759548 34955979 PMC8692942

[45] Xing Y, Starik M. Taoist leadership and employee green behaviour: A cultural and philosophical microfoundation of sustainability. Journal of Organizational Behavior. 2017;38(9):1302–19.

[46] Arici HE, Uysal M. Leadership, green innovation, and green creativity: A systematic review. The Service Industries Journal. 2022;42(5–6):280–320.

[47] Chen J, Ghardallou W, Comite U, Ahmad N, Ryu HB, Ariza-Montes A, et al. Managing hospital employees’ burnout through transformational leadership: the role of resilience, role clarity, and intrinsic motivation. International Journal of Environmental Research and Public Health. 2022;19(17):10941. doi: 10.3390/ijerph191710941 36078657 PMC9518422

[48] Du Y, Yan M. Green transformational leadership and employees’ taking charge behavior: The mediating role of personal initiative and the moderating role of green organizational identity. International Journal of Environmental Research and Public Health. 2022;19(7):4172. doi: 10.3390/ijerph19074172 35409857 PMC8998811

[49] Chen C, Rasheed A, Ayub A. Does Green Mindfulness Promote Green Organizational Citizenship Behavior: a Moderated Mediation Model. Sustainability. 2023;15(6):5012.

[50] Talebzadeh N, Nosrati S, Rezapouraghdam H, Naderi S, Kilic H, Karatepe OM. Green Transformational Leadership, Green Work Engagement and Their Impacts on Hotel Employees’ Green Behaviors: A Moderated-Mediation Model. Co-Editors. 2022;33.

[51] Guo M, Ahmad N, Adnan M, Scholz M, Naveed RT. The relationship of csr and employee creativity in the hotel sector: The mediating role of job autonomy. Sustainability. 2021;13(18):10032.

[52] De Young R. New ways to promote proenvironmental behavior: Expanding and evaluating motives for environmentally responsible behavior. Journal of social issues. 2000;56(3):509–26.

[53] Ericson T, Kjønstad BG, Barstad A. Mindfulness and sustainability. Ecological Economics. 2014;104:73–9.

[54] Vallerand RJ, Salvy SJ, Mageau GA, Elliot AJ, Denis PL, Grouzet FM, et al. On the role of passion in performance. Journal of personality. 2007;75(3):505–34. doi: 10.1111/j.1467-6494.2007.00447.x 17489890

[55] Robertson JL, Barling J. Greening organizations through leaders’ influence on employees’ pro‐environmental behaviors. Journal of organizational behavior. 2013;34(2):176–94.

[56] Jia J, Liu H, Chin T, Hu D. The continuous mediating effects of GHRM on employees’ green passion via transformational leadership and green creativity. Sustainability. 2018;10(9):3237.

[57] Peng J, Chen X, Zou Y, Nie Q. Environmentally specific transformational leadership and team pro-environmental behaviors: The roles of pro-environmental goal clarity, pro-environmental harmonious passion, and power distance. Human Relations. 2021;74(11):1864–88.

[58] Afsar B, Badir Y, Kiani US. Linking spiritual leadership and employee pro-environmental behavior: The influence of workplace spirituality, intrinsic motivation, and environmental passion. Journal of Environmental Psychology. 2016;45:79–88.

[59] Kura KM. Linking environmentally specific transformational leadership and environmental concern to green behaviour at work. Global Business Review. 2016;17(3_suppl):1S–14S.

[60] Tosun C, Parvez MO, Bilim Y, Yu L. Effects of green transformational leadership on green performance of employees via the mediating role of corporate social responsibility: Reflection from North Cyprus. International Journal of Hospitality Management. 2022;103:103218.

[61] Obeng AF, Zhu Y, Azinga SA, Quansah PE. Organizational climate and job performance: Investigating the mediating role of harmonious work passion and the moderating role of leader–member exchange and coaching. Sage Open. 2021;11(2):21582440211008456.

[62] Choong Y-O, Ng L-P, Tee C-W, Kuar L-S, Teoh S-Y, Chen I-C. Green work climate and pro-environmental behaviour among academics: The mediating role of harmonious environmental passion. International Journal of Management Studies. 2019;26(2):77–97.

[63] Ari E, Karatepe OM, Rezapouraghdam H, Avci T. A conceptual model for green human resource management: Indicators, differential pathways, and multiple pro-environmental outcomes. Sustainability. 2020;12(17):7089.

[64] Liu Y, Ahmad N, Lho LH, Han H. From boardroom to breakroom: Corporate social responsibility, happiness, green self-efficacy, and altruistic values shape sustainable behavior. Social Behavior and Personality. 2024;52(2).

[65] Xu L, Cherian J, Zaheer M, Sial MS, Comite U, Cismas LM, et al. The role of healthcare employees’ pro-environmental behavior for De-carbonization: an energy conservation approach from CSR perspective. Energies. 2022;15(9):3429.

[66] Iyer P, Davari A, Paswan A. Green products: Altruism, economics, price fairness and purchase intention. Social Business. 2016;6(1):39–64.

[67] Bautista R, Dui R, Jeong LS, Paredes MP. Does altruism affect purchase intent of green products? A moderated mediation analysis. Asia-Pacific Social Science Review. 2020;20(1):159–70.

[68] Tu Y, Li Y, Zuo W. Arousing employee pro‐environmental behavior: A synergy effect of environmentally specific transformational leadership and green human resource management. Human Resource Management. 2023;62(2):159–79.

[69] Bass BM, Riggio RE. Transformational leadership, 2nd ed. Mahwah, NJ, US: Lawrence Erlbaum Associates Publishers; 2006. xiii, 282–xiii, p.

[70] Ahmad N, Ahmad A, Siddique I. Beyond self‐interest: how altruistic values and human emotions drive brand advocacy in hospitality consumers through corporate social responsibility. Corporate Social Responsibility and Environmental Management. 2023.

[71] Ahmad N, Samad S, Han H. Travel and Tourism Marketing in the age of the conscious tourists: a study on CSR and tourist brand advocacy. Journal of Travel & Tourism Marketing. 2023;40(7):551–67.

[72] Robertson JL. The nature, measurement and nomological network of environmentally specific transformational leadership. Journal of Business Ethics. 2018;151(4):961–75.

[73] Blok V, Wesselink R, Studynka O, Kemp R. Encouraging sustainability in the workplace: A survey on the pro-environmental behaviour of university employees. Journal of cleaner production. 2015;106:55–67.

[74] Schwartz SH. Universals in the content and structure of values: Theoretical advances and empirical tests in 20 countries. Advances in experimental social psychology. 25: Elsevier; 1992. p. 1–65.

[75] Daniel S. A-priori Sample Size Calculator for Structural Equation Models 2010 [May 28, 2022].

[76] Fu B, Ahmad N, Lho LH, Han H. Triple-E effect: Corporate ethical responsibility, ethical values, and employee emotions in the healthcare sector. Social Behavior and Personality: an international journal. 2023;51(12):12735E–48E.

[77] Liu Y, Cherian J, Ahmad N, Han H, de Vicente-Lama M, Ariza-Montes A. Internal corporate social responsibility and employee burnout: an employee management perspective from the healthcare sector. Psychology Research and Behavior Management. 2023:283–302. doi: 10.2147/PRBM.S388207 36761414 PMC9904231

[78] Ahmad N, Ullah Z, AlDhaen E, Han H, Ariza-Montes A, Vega-Muñoz A. Fostering advocacy behavior of employees: A corporate social responsibility perspective from the hospitality sector. Frontiers in Psychology. 2022;13:865021. doi: 10.3389/fpsyg.2022.865021 35572254 PMC9093048

[79] Fu Q, Cherian J, Ahmad N, Scholz M, Samad S, Comite U. An inclusive leadership framework to foster employee creativity in the healthcare sector: the role of psychological safety and polychronicity. International journal of environmental research and public health. 2022;19(8):4519. doi: 10.3390/ijerph19084519 35457388 PMC9028499

[80] Han H, Al-Ansi A, Chua B-L, Ahmad N, Kim JJ, Radic A, et al. Reconciling civilizations: Eliciting residents’ attitude and behaviours for international Muslim tourism and development. Current Issues in Tourism. 2023;26(9):1463–81.

[81] Ahmad N, Ullah Z, AlDhaen E, Han H, Araya-Castillo L, Ariza-Montes A. Fostering hotel-employee creativity through micro-level corporate social responsibility: a social identity theory perspective. Frontiers in psychology. 2022;13:853125. doi: 10.3389/fpsyg.2022.853125 35572307 PMC9093142

[82] Zhou X, Ahmad N, Lho LH, Han H. Social ripple: Unraveling the impact of customer relationship management via social media on consumer emotions and behavior. Social Behavior and Personality: an international journal. 2023;51(10):1–12.

